# The influence of ethnic minority background and migration history on recovery in psychotic disorders: A systematic literature review

**DOI:** 10.1192/j.eurpsy.2021.358

**Published:** 2021-08-13

**Authors:** L. Pasinelli, A. Bakia, I. Tarricone, C. Mulder, J.-P. Selten

**Affiliations:** 1 Department Of Medical And Surgical Sciences, Section Of Psychiatry, University of Bologna, Bologna, Italy; 2 Department Of Psychiatry, Epidemiological And Social Psychiatric Research Institute, Erasmus MC, Rotterdam, Netherlands; 3 Department Of Psychiatry Andneuropsychology, School For Mental Health And Neuroscience, South Limburg Mental Health Research Andteaching Network, Maastricht University Medical Centre, Maastricht, Netherlands

**Keywords:** Psychotic disorders, recovery, migrants, ethnic minorities

## Abstract

**Introduction:**

Recovery in psychotic disorders is a concept that evolved through the last decades. Thanks to the contribution of different researchers, together with the recovery movement, a switch happened from a service-based to a client-based approach towards recovery. The Dutch framework considers recovery as the interplay of symptomatic, personal, functional and societal aspects, determined by different biological, psychological, personal and social factors. Literature on this fourdimensional perspective is still scarce. In addition, even if an increased incidence of psychotic disorders has been recognized in ethnic minority populations and migrants, studies on the influence of ethnicity and migration on recovery in psychotic disorders is limited.

**Objectives:**

To write a systematic literature review on how ethnic minority status and migration history may affect symptomatic, personal, functional and societal recovery.

**Methods:**

A systematic search of the main databases, followed by a four-step selection process to include studies comparing migrants or ethnic minority populations and the non-minoritarian/autochthonous population in terms of recovery. A qualitative, narrative summary has been performed.

**Results:**

Thirty-eight articles have been included. Literature is heterogeneous, focused on clinical outcomes and mostly based on data from the UK and the USA. As a common thread, ethnic minority status and migration history result to negatively influence societal, personal and, to a lower extent, clinical recovery.

**Conclusions:**

Further studies based in different cultural backgrounds and focused on recovery in its multiple aspects are needed, to get a better understanding of the contextual and structural factors that affect the interaction between ethnicity, migration and recovery in psychotic disorders.

**Disclosure:**

No significant relationships.

